# Analysis of Motor Intervention Program on the Development of Gross Motor Skills in Preschoolers

**DOI:** 10.3390/ijerph17134891

**Published:** 2020-07-07

**Authors:** Cecilia Ruiz-Esteban, Jaime Terry Andrés, Inmaculada Méndez, Ángela Morales

**Affiliations:** 1 Department of Evolutionary and Educational Psychology, Campus Regional Excellence Mare Nostrum, University of Murcia, 30100 Murcia, Spain; cruiz@um.es (C.R.-E.); jaime.terry@um.es (J.T.A.); 2 Department of Music, Autonomous University of Madrid, 28049 Madrid, Spain; angela.morales@uam.es

**Keywords:** physical activity, motor development, motor activity program, preschool education

## Abstract

This study aimed to investigate the influence of a structured movement activity program on the motor development of children aged three to five years attending preschool. Participants were 136 preschool students with normative development at three to four years old who lived in the Region of Murcia (Spain). The McCarthy Children’s Psychomotricity and Aptitude Scales (MSCA) battery of psychomotor tests was used to evaluate the motor development profiles of preschoolers before and after the intervention. The sample was divided into two groups: an intervention group (28 students) and a comparison group (108 students). A structured 24 week physical education program was used in the intervention group. An experiential program based on free play was used in the comparison group during the same period. Preschoolers in both groups got a significant improvement in the contrast of pre-intervention with post-intervention in limb coordination. Statistically significant differences in the post-intervention measurements between the comparison group and the intervention group on arm and leg coordination were observed, whereby the intervention group presented higher arm coordination values (*F*_1,134_ = 14,389, *p* = 0.000, *η*^2^ = 0.097) and higher leg coordination values (*F*_1,134_ = 19,281, *p* = 0.000, *η*^2^ = 0.126) than the comparison group. It was pointed out that structured physical activity education is better educational methodology than free play to achieve adequate motor development in preschool children.

## 1. Introduction

Physical stimulation is essential for progressive acquisition of the mental and motor skills that determine children’s development [1]. An active lifestyle during childhood is beneficial for physical, cognitive, and mental health [2]. Child psychomotor development seeks to provide children with the necessary skills to guarantee good school achievement [3,4]. Hraste et al. [5] examined the efficiency of an integrated mathematics/geometry and physical activity program and found its effectiveness. In addition, Padulo et al. [6] found that fundamental motor skills such as jump or run correlated to cognitive achievement in areas such as English, mathematics, sport, and technologies.

The physical opportunities offered at school and at home are important settings for improving children’s physical and psychological-cognitive status [6] and for facilitating development. Campos et al. [7] showed the similarity in motor progress in children in the first months of schooling; however, this diverges as children grow older. Thus, a school must create appropriate learning opportunities [8] for children to experience different motor practices in suitable places, equipped with specialized materials, and it must be prepared to carry out specific educational activities that take into account the individual characteristics and needs of children. According to McClenaghan and Gallahue [9], gross psychomotricity, or psychomotor behaviors based on general coordination, describes the fundamental patterns of movement. These patterns include coordination of the arms and legs. At this age, specific educational activities should focus on limb coordination.

Free play is often the only opportunity children have to participate in movement activities, and while this may encourage movement, it does not promote learning of fundamental movement skills [1]. Although the term development may imply that motor development competence is acquired "naturally" through maturation processes, this does not happen [2]. These skills must be learned, practiced, and reinforced [3,4]. Motor skills practices should consist on planned movement activities that are specific to development and instruction.

During preschool, both an encouraging environment and the participation of children in motor activities redound in normal development and affect the general health of children [10]. The progressive acquisition of skills related to mental and motor activities is defined as psychomotor development [11]. If gross motor development is not mastered, children may experience lifelong difficulties in acquiring later motor skills. Therefore, the acquisition of gross motor skills is critical, but despite this, many preschool teachers have overlooked the importance of gross motor development [12]. Thus, teachers must create appropriate learning opportunities [8] for children to experience different motor practices in suitable places, equipped with specialized materials, and they must be prepared to carry out specific educational activities that take into account the individual characteristics and needs of children.

In this sense, Palma [13] carried out a study with 95 preschoolers in which the difference between an experiential program (enriched free play) and a structure program based on a combination of exploration, free play, oriented play, and activities was studied (guided oriented game). Children’s participation in the oriented play program had a positive effect on their motor development, while no changes were observed in either the free play group in an enriched environment or the comparison group. Logan et al. [14], in meta-analyses, pointed out motor skill interventions are effective in improving motor development in preschoolers. Kindergarten should implement structured movement programs as a strategy to promote motor skill development in children. Thus, it is important to promote continuous learning and the development of motor competence through participation in planned movement interventions [4,14,15].

There is no unanimity regarding the type of program to be used for the development of motor skills [16]. Program used may vary from the free play (nondirective or experiential type) to oriented activities (or functional type). Therefore, there is a need for research to determine the effectiveness of movement program-based interventions in early education settings [14,17,18]. However, there are few studies on these programs in preschool. Giagazoglou et al. [19] found that a 12 week, motor intervention program improved gross as well as fine motor skills. Along these same lines are the studies by Kambas et al. [20], Terry [17], and de Zimmer et al. [21].

Iivonena and Sääkslahti [22], in a systematic review, founded a series of studies that included pre-test and posttest comparisons of educational intervention in preschool children. The duration of the programs ranged from eight weeks to eight months. In general, the main objectives of the programs were related to the improvement of motor skills and physical activity of children, although they used different methodologies and obtained different results. Jones et al. [23] conducted a study to assess the efficacy of a 24 week movement skills development physical activity program that included three structured 20 min lessons per week and teacher training sessions. The program improved the overall movement and jumping skill scores. Reilly et al. [24] noted that improved 24 week physical activity with three 30 min lessons per week plus nutritional training for families to reduce children’s BMI improved fundamental movement skills scores. In an international project by Iivonena et al. [22], they used an eight month physical education curriculum that included two 45 min lessons per week that improved boys’ running speed and wide foot jump in girls. The Munch and Move program (PMENT) [25] aimed at healthy eating, physical activity, and movement skills to improve points on the locomotive subtest, object control subtest, and total score of fundamental motor skills. Deli et al. [26] developed an eight week music and movement program with critical education that included two 35 min lessons per week implemented by a physical activity teacher, and this improved jumping and dynamic balance.

As Logan et al. [14] indicates, it is important that researchers continue to investigate the type of interventions, the approach, and the instructional time to determine the optimal movement program. Researchers are encouraged to investigate and report all intervention studies in this area. The aim of this study was to determine the influence of structured a physical education program on the psychomotor development of three- to four-year-old preschool students by identifying, describing, and comparing the psychomotor skill variations of an intervention group in contrast with a comparison group. Particularly, this study was to determine the differences in the development of leg and arm coordination, using a structured motor intervention compared with the free play program, in boys and girls attending preschool aged 34 to 44 months.

## 2. Materials and Methods

### 2.1. Participants

Participants were 136 students of early childhood education centers in the Region of Murcia. Their ages at the start of the intervention ranged from 34 to 44 months when the first measure was applied. The mean age and standard deviation of the intervention group was 38.39 (±3.30) months and the comparison group was 39.5 (±3.00) months. Of all participants, 48.5% were male. The preschool children were distributed among the intervention and comparison groups as follows in Table 1.

This was an intentional nonprobabilistic sample. To avoid the effect that different teachers may have on preschool psychomotricity learning, a sample was chosen in which the same teacher could teach the intervention group. The comparison group’s teachers were assigned to each group and were kept throughout the study to control possible methodological effects due to the reactive effect of introducing another observer in a natural context or the experimenter/observer effect, which could cause distortions in the study. The inclusion criteria were as follows: (a) children in first grade of early childhood education; (b) not having diagnosed motor development problems; (c) who have received parental authorization.

A total of 140 children were recruited. Applying the inclusion and exclusion criteria, four subjects were not included. Of the 136 participants, 28 children made up the intervention group and 108 the comparison group.

### 2.2. Instruments

Two of the McCarthy Children’s Psychomotricity and Aptitude Scales (MSCA, USA) were applied [27]. The scales used to assess gross motor skills were (a) leg coordination scale including six items, which includes walking backwards, walking on tiptoe, walking on a straight line, staying on the right foot, staying on the left foot, and rhythmically jumping on either foot (without repeatedly resting on the same foot). There are two attempts in each one. The first five items are scored from 0 to 2 and the sixth from 0 to 3. The maximum score of this test is 13; (b) arm coordination including bounce a ball (scored 0 to 7, it is assessed if the child is able to bounce the ball from 0 to more than 15 times with any arm); catch a beanbag (catch the beanbag with two hands scores 0 to 3; only with one hand scores 0 to 3 each arm. Total item is scored 0 to 9); target shooting (scored 0 to 12. Three attempts are made with each arm). The maximum scored in Arm Coordination Scale is 28. In this study, Cronbach’s alpha coefficient was 0.762 for legs coordination scale and 0.863 for arms coordination scale. The Spanish adaptation by *TEA Ediciones* was used in this study [28].

The population scale of the subtest was not used, since Cortadellas [29] affirmed, after carrying out an analysis, that it has a ceiling effect on several subscales.

### 2.3. Procedure

After obtaining the necessary permits from school authorities, the researchers informed the parents of the preschoolers about the objectives of the study and the instruments used, who then authorized their child’s participation voluntarily and anonymously, and their responses were kept confidential.

The intervention was carried out in the regular movement activity schedule with preschoolers. As the school randomly assigned the children to the different groups, two of the groups were chosen at random to be an intervention group and the rest were elected as a comparison group [30]. This study used a pre-test–post-test quasi-experimental design.

Chi squared was performed to confirm the homogeneity of the samples among the intervention group and the comparison group. Comparison of samples for leg coordination (*p* = 0.133) and for arm coordination (*p* = 0.394) confirmed the homogeneity of the sample prior to the intervention.

Data collection was carried out in two stages, one prior to the application of the program intervention (test) and another after the intervention finished. To carry out the data registration, we analyzed the determining factors (illness, excitement, accidents during assessment [31]) that should be taken into account to ensure the reliability of the tests. This procedure of analysis and control of the determining factors was carried out with the objective of not affecting the variables by using the same conditions in all cases. In this way, we ensured that the procedure of analysis used did not affect the results of the investigation.

Seven conditioning aspects that needed to be controlled throughout the process were defined:

1. The schedule of the intervention.
2. The collection, registration process, and delivery methods used.
3. The space where the materials used to carry out the registration were located.
4. The environmental situation of the space, e.g., sound, temperature, lighting, comfort.
5. The materials used to proceed with the tests and their features.
6. The duration of each registration.
7. The distribution of children by groups and classes.

The intervention that was carried out during the research project was based on different educational methodologies used in psychomotricity classes. After the first MSCA tests, different programs corresponding to the intervention and comparison groups were applied. The intervention group included two scholar groups of preschoolers (infants were from 34 to 44 months old) chosen at random, corresponding to two classes of first-grade students. The remaining four classes (same age and grade) formed the comparison group. Children were assigned to classroom randomly by the school principal. Three teachers participated in the study. One taught both intervention groups and the other two taught the comparison group. Teacher assignation to each group was also random.

**Figure 1:**
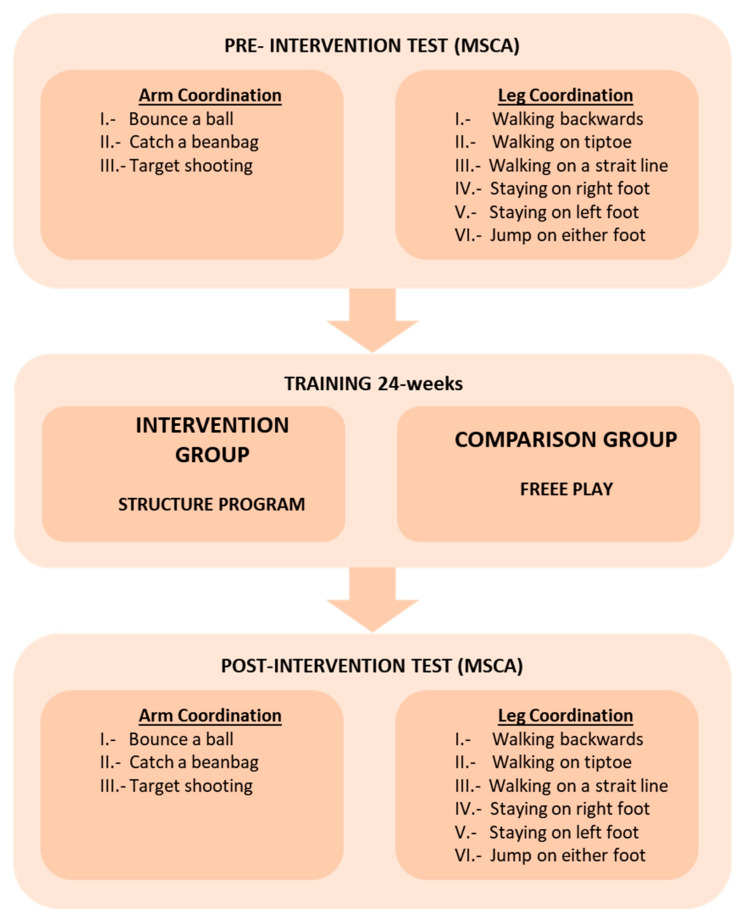
Procedure model.

The intervention consisted of an educational psychomotor program that was implemented to improve motor development. The training program for each group was applied twice a week for 24 weeks, and each session lasted 45 min (See Figure 1).

Intervention group. Participants in this group received an intervention developed from a preschool physical education curriculum developed by Terry [17] based on studies by Corrales et al. [32] and Vaca [33]. The goal of the program was to elicit improvement in fundamental motor skills via appropriate instruction and practice. This intervention focused on the motor and cognitive aspects. Three cognitive and motor areas were fundamentally organized: body schema, spatial schema, and temporal schema. These schemes were being addressed in parallel, the body schema being the basis for the elaboration of the spatial schema, and the protocols in turn serving as the basis for the construction of the temporal schema. The sessions design was based on three aspects: (a) organization of the body scheme (body perception and control, postural balance, breathing); (b) basic motor behaviors (balance, general dynamic coordination, visual-manual coordination); (c) neuromotor behaviors (paratonia, synkinesis, laterality, perceptual-motor behaviors, spatial organization, rhythm and motor activity, time organization and structuring). Each session had seven phases:

- Presentation (pick up the group in their classroom and travel to the gym or playground);
- Communication and instruction (assembly, sit down to explain the session, communicate rules, raise awareness);
- Organization of space and materials (presentation of space, limits, areas, the corners, materials, the possibilities of action, the actions not allowed, the safe attitudes);
- Animation and exploration (let them inspect the space and materials, provide stimuli to approach the corners, facilitate the use of all spaces and materials, motivate the use of resources);
- Application (it is the most sensitive phase in its implementation in action, since it introduces the mechanisms of intervention);
- Communication (phase of collection, recovery, relaxation, in which the teacher exchanges comments with the students, motivates them to communicate and interpret feelings and experiences);
- Farewell (back to the classroom, talk to the tutor about achievements, what they have done and involve all children, small assembly related to the session).

During application phase, the activity focused on motor skills such as walking, running, jumping, rolling, sliding, galloping, leaping, striking, dribbling, kicking, throwing, and catching in different levels of achievement. Participants followed a movement training routine using a variety of gym and playground equipment. Each session was challenging and motivating to preschoolers.

Comparison group. Participants in comparison group received a typical kindergarten activity program involving free play. Sessions consisted of indoor and outdoor free play. Free play consisted on supervised play on the playground or gym using hoops, steps, balls, bats, and other usual material.

One teacher (20 years of experience) taught both intervention groups. This teacher was also the program developer. Two other teachers taught the comparison groups (15 and 22 years of experience, respectively). They did not receive any training because free play was the regular physical activity program in this school.

None of the children in the comparison or intervention group participated in other organized physical activities during the intervention.

The study protocol was approved by the Ethics Committee for Clinical Research of the University of Murcia (ID: 2478/2019). This study was conducted in accordance with the approved guidelines and the Declaration of Helsinki.

### 2.4. Data Analysis

Chi-squared was used to determine the homogeneity of two groups among the initial measurements. Separate 2 × 2 factorial MANOVA (Multivariate analysis of variance) evaluated the interaction effect between experimental condition (Intervention Group vs. Comparison Group) and time (pre and post testing), with respect to the legs and arm coordination of preschoolers. In addition, Snedecor’s *F* distribution was calculated as a continuous probability distribution that arises as the null distribution of a test statistic in the analysis of variance, and Eta Squared (*η*^2^) values were also used to control effect size. The level of significance was set at *p* < 0.05. For the data analysis, the statistical program SPSS v.23 [34] was used.

## 3. Results

Table 2 shows the means and standard deviations of limb coordination variables obtained from the initial measurements (pre-test) and the final measurements (post-test) of the participants under study who carried out the structured motor intervention.

After applying an analysis of variance of two factors (2 × 2), the structures motor intervention and the moment of measurement, with repeated measures in the last factor, we observed that the effect of the interaction of the moment of measurement factor with the structures motor intervention was significant for leg coordination (*F*_1,134_ = 18,178, *p* = 0.000, *η*^2^ = 0.119). The effect of the interaction of the moment of measurement factor with the structures motor intervention was also significant for arm coordination (*F*_1,134_ = 40.946; *p* = 0.000, *η*^2^ = 0.234). Therefore, it can be stated that the interaction between both factors affects the changes produced in limb coordination.

From the perspective of the inter-subject factor (structures motor intervention), the initial measurement (pre-test) of leg coordination was slightly higher in the comparison group than in the intervention group, although there were no statistically significant differences (*F*_1,134_ = 0.006, *p* = 0.938, *η*^2^ = 0.000). The initial measurement (pre-test) of arm coordination produced higher values in the comparison group than in the intervention group, although no statistically significant differences were observed (*F*_1,134_ = 1.804, *p* = 0.182; *η*^2^ = 0.013).

**Figure 2:**
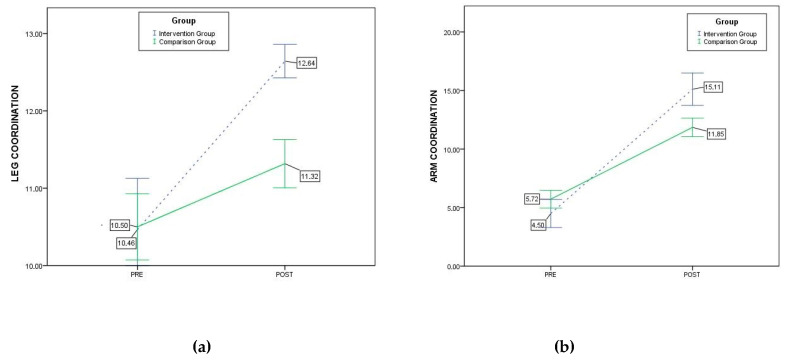
Evolution of leg coordination (**a**) and arm coordination (**b**) related to the intervention and comparison groups.

When analyzing the evolution of the values obtained for leg coordination (Figure 2a), statistically significant increases were observed both in the intervention group (*F*_1,134_ = 57.307, *p* = 0.000, *η*^2^ = 0.300) and in the comparison group (*F*_1,134_ = 29.931, *p* = 0.000, *η*^2^ = 0.183). When analyzing the evolution of the values obtained for arm coordination (Figure 2b), statistically significant increases were observed in both the intervention group (*F*_1,134_ = 289.364, *p* = 0.000, *η*^2^ = 0.683) and the comparison group (*F*_1,134_ = 372.720, *p* = 0.000, *η*^2^ = 0.736). Therefore, analyzing the evolution of the values, the intervention group performed significantly better than the comparison group from pre-test to post-test for both leg coordination and arm coordination.

Finally, when analyzing the differences in the post-intervention measurements between the comparison group and the intervention group, statistically significant differences were observed (*F*_1,134_ = 19,281, *p* = 0.000, *η*^2^ = 0.126), whereby the intervention group presented higher leg coordination values than the comparison group. Analyzing the differences in the post-intervention measurements between the comparison group and the intervention group on arm coordination, statistically significant differences were observed (*F*_1,134_ = 14,389, *p* = 0.000, *η*^2^ = 0.097), whereby the intervention group presented higher arm coordination values than the comparison group. The small value of the effect size in the post intervention measures indicates that data should be taken with caution due to the sample size.

## 4. Discussion

Campos et al. [7] pointed out the similarity among children in motor progress in the first months of schooling, which diverges as children grow older. In line with our research, the data were homogeneous between groups prior to the intervention. Due to development, each three-year-old child improves motor skills. In this line, according to our findings, preschoolers in the comparison group got a significant improvement in the contrast of pre-intervention with post-intervention in both arm and leg coordination.

There is a false belief that children are spontaneously active [11], but children who are not directed in the practice of motor activities may have slower development in their abilities [35,36]. Along the same lines as indicated in our study, the research carried out by Bundy et al. [37], Favazza et al. [38], Palma [13], and Terry [17] indicated that a structured program of classes based on physical education for preschoolers helps to initiate their physical activity and increase their motor development [39,40,41]. The results of the present study coincide with the conclusions of Costa et al. [42], Silva, Almeida and Moreira [43], and Teixeira et al. [11], who argued that a structured program of physical activities has a great impact on the development of motor skills in preschoolers. However, they do not coincide with those found by Jambunathan [44] and Valentini and Rudisill [3], who argued that the best intervention programs to improve children’s motor development (autonomy and quality of movement) were those based on free activities. In addition, according to Simó and Espada [45], in the teachers’ opinion, children should have freedom and autonomy, so that they can fully develop motor skills.

Literature suggests that fundamental motor skills competences increase more in preschool ages. The review by Iivonena and Sääkslahti [22] gave support to that evidence. These findings suggest that various types of preschool programs that include the goal of developing motor skills, lasting eight weeks to eight months, and implementing at least two structured sessions per week can improve children’s fundamental motor skills and may help to involve locomotor skill development such as limb coordination and balance. In this sense, this research has used a 24 week intervention with three weekly sessions based on a structured program, which has improved fundamental motor skills such as jumping, balancing, or throwing.

Jonas et al. [23] applied the JUMP START program to an intervention group during 20 weeks. The control group continued with their usual curriculum, which included free play. They reported a significant improvement in five movement skills (run, catch, jump, kick, and hop) in the intervention group. They concluded that the structured lesson allowed focus on those components that children found more difficult to master. For instance, mastery of the jump requires coordinated movement between the arms and the legs which young children often find difficult. However, with direct instruction and adequate practice time, mastery can potentially be accomplished. In this study, with a similar intervention period and classes based on free play for the control group, structured lessons could have made teachers be more involved in instruction than when the curriculum implies only free play. Therefore, likely limb coordination, which is a difficult fundamental motor skill to master by preschoolers, was more intensively taught to the intervention group than to those in the comparison group.

Goodway, Crowe, and Ward [46] analyzed a nine-week instructional program on locomotor skill development of 33 preschoolers who were at risk. This study compared the effect of motor skills intervention on the development of fundamental motor skills, and the performance of the intervention group was significantly better after the SKIP program (Successful Kinesthetic Instruction for Preschoolers) than that of the comparison group before and after the test for locomotor and object control skills. There are important differences in the program itself and the instrument used between the Goodway et al. [46] study and this research, but there are also similarities. After a well-structured program, functional development of motor skills improves more than after a free play program.

As Lemos et al. [47], after a study with an intervention lasting a full academic year to 25 children in each group (intervention and control), pointed out, gross motor development is better if a physical activity specialist teaches it than if it is a regular preschool teacher who provides movement activities education. Thus, specialists should teach a preschool motor program or a preschool teacher should be trained on this area because structured practice and appropriate instruction are crucial in promoting gross motor development, especially skills such as limb coordination and balance [18].

Although the scale provided by the MSCA on leg coordination and arm coordination has been taken as a reference in this study, both scales include a good number of fundamental motor skills such as balance, jump, or bounce. Actually, the structured program has been better than the free game in the development of tasks such as walking, static balance, dynamic balance, and jump in regards to the mastery of leg coordination; and it has also been better at bouncing, catching, and throwing in terms of arm coordination. All of them are complex activities for children from three to four years old. More structured activities should be incorporated into the preschoolers’ curriculum instead of free play sessions to promote preschool motor development.

There are many obstacles for preschool centers to offer good gross motor skills development programs. First, preschoolers need adequate equipment for their age and body size. Second, sufficient play space is required for movement activities. Not all educational centers have adequate space to have an intervention environment. Third, early childhood teachers may not be trained to implement the best movement skill program.

From the research point of view, it is important to continue determining the most effective characteristics of motor skills procedures (i.e., minutes of instructional time, instructional approaches) to shape policy recommendations and study plans. Structured movement programs in early childhood settings are a better educative intervention to facilitate motor development than free play. In addition, training in coordination increases intelligence quotient [48].

These findings should be taken with caution due to a number of limitations. The first limitation of this study is that the sample is not representative and therefore data are not generalizable. As a pilot study, data serve as a starting point for other investigations with a larger number of participants. Other limitations of this study include the difficulty of applying the MSCA motor scale. Kron and Traxler [49] and Eiser [50] presented evaluations about the scarcity of studies that refer to the discrimination of different subtests by age and sex. Similarly, Cortadellas [29] stated that, in general, studies on the McCarthy instrument refer to scales, or some refer to subtests, but in no case does an analysis refer to the discriminative value of the items. On the other hand, in the review carried out by Scheuer, Herrmann, and Bund [51] on the use of instruments to assess gross psychomotricity in infant and primary education, only three scales were detected for children of the same ages used this study. The MoTB (Motor Test Battery 3-7) was used by Krombholz [52] in children aged from three to seven years old, the TGMD-2 (Test of Gross Motor Development-2nd Edition) was used by Ulrich [53] in children aged from three to eleven years old, and the PDMS-2 (Peabody Developmental Motor Scale) was used by Folio and Fewell [54] for children aged from birth to seven years old. However, more validation studies are needed to improve the psychometric quality of the existing instruments, especially in an educational context. Longer longitudinal studies are also necessary to discriminate the benefits of both pedagogical methods [51]. Furthermore, due to the sample size and the design of the intervention, several teachers have intervened, however, teachers’ fidelity has not been studied. Finally, the value of the effect size in the post-intervention measures indicates that we should take these data with caution.

## 5. Conclusions

This study’s findings support the limited literature suggesting that directed motor skill instruction has a positive impact on fundamental motor skill development.

The results of this study indicate the supremacy of the directed movement program over the free play program on fundamental motor skills development such as leg and arm coordination. According to our findings, increasing the presence of psychomotor intervention based on structured sessions versus free play in preschool is recommended to better develop limb coordination. Therefore, more and better training should be given to teachers of infants in physical activity in order to optimize the development of their preschool-aged students.

## Figures and Tables

**Table 1:** Sample distribution.

	Participants					
	Males		Females		Total	
	*n*	%	*n*	%	*n*	%
Total	66	48.5	70	51.5	136	100
Intervention group	14	10.3	14	10.3	28	20.6
Comparison group	52	38.2	56	41.2	108	79.4

**Table 2:** General descriptive data on leg and arm coordination.

		Pre-Test		Post-Test	
		Mean	Standard Deviation	Mean	Standard Deviation
**Leg Coordination**	Intervention Group	10.46	1.71	12.67	0.47
	(*n* = 28)				
	Comparison Group	10.50	2.24	11.31	1.62
	(*n* = 108)				
**Arm Coordination**	Intervention Group	4.50	3.09	15.10	3.56
	(*n* = 28)				
	Comparison Group	5.72	3.97	11.85	4.15
	(*n* = 108)				
